# Suppression of starch synthase I expression affects the granule morphology and granule size and fine structure of starch in wheat endosperm

**DOI:** 10.1093/jxb/eru095

**Published:** 2014-03-14

**Authors:** Stephen J. McMaugh, Jenny L. Thistleton, Emma Anschaw, Jixun Luo, Christine Konik-Rose, Hong Wang, Min Huang, Oscar Larroque, Ahmed Regina, Stephen A. Jobling, Matthew K. Morell, Zhongyi Li

**Affiliations:** ^1^CSIRO Food Future Flagship, GPO Box 1600, Canberra, ACT 2601, Australia; ^2^CSIRO Division of Plant Industry, GPO Box 1600, Canberra, ACT 2601, Australia

**Keywords:** Endosperm, expression, starch granule morphology, starch structure, starch synthase I, wheat.

## Abstract

Studies in *Arabidopsis* and rice suggest that manipulation of starch synthase I (SSI) expression in wheat may lead to the production of wheat grains with novel starch structure and properties. This work describes the suppression of SSI expression in wheat grains using RNAi technology, which leads to a low level of enzymatic activity for SSI in the developing endosperm, and a low abundance of SSI protein inside the starch granules of mature grains. The amylopectin fraction of starch from the SSI suppressed lines showed an increased frequency of very short chains (degree of polymerization, dp 6 and 7), a lower proportion of short chains (dp 8–12), and more intermediate chains (dp 13–20) than in the grain from their negative segregant lines. In the most severely affected line, amylose content was significantly increased, the morphology of starch granules was changed, and the proportion of B starch granules was significantly reduced. The change of the fine structure of the starch in the SSI-RNAi suppression lines alters the gelatinization temperature, swelling power, and viscosity of the starch. This work demonstrates that the roles of SSI in the determination of starch structure and properties are similar among different cereals and *Arabidopsis*.

## Summary

Manipulation of SSI expression in wheat using RNAi technology leads to the production of wheat grains with novel starch structure and properties.

## Introduction

Starch is the major component of wheat grain (approximately 60–70%). Starch granules in higher plants contain two main classes of glucan polymer, amylose and amylopectin. The amylose fraction of wheat starch is a linear or infrequently branched (containing <1% α-1,6 linkages) molecule with a degree of polymerization (dp) of 500–3000. Amylopectin is a large and highly branched polymer (containing 5–6% α-1,6 linkages) reaching a dp of about 5000–50 000 (see reviews from Kossmann and Lloyd, 2000; Rahman *et al.*, 2000; Ball and Morell, 2003; James *et al.*, 2003; Tetlow *et al.*, 2004; Morell *et al.*, 2006). The ratio of these two polymers and the fine structure of amylopectin influence the processing, cooking, organoleptic qualities, and digestibility of starch-based foods.

Starch polymers are produced by a suite of enzymes including ADP-glucose pyrophosphorylases (EC 2.7.7.27), starch synthases (EC 2.4.1.21), starch-branching enzymes (EC 2.4.1.18), disproportionating enzymes (EC 2.4.1.25), and starch-debranching enzymes (EC 3.2.1.41 and 3.2.1.68) (see reviews from Kossmann and Lloyd, 2000; Rahman *et al.*, 2000; Ball and Morell, 2003; James *et al.*, 2003; Tetlow *et al.*, 2004; Morell *et al.*, 2006). ADP-glucose pyrophosphorylases catalyse the first committed step of starch synthesis in plant tissues by synthesizing ADP-glucose, the activated glucosyl precursor, as the source of glucose for starch elongation and subsequently branching. Starch synthases are involved in the elongation of the glucan chains through the addition of glucose residues from ADP-glucose at the non-reducing end of the glucan chains. The α-1,6 linkages within starch are introduced by starch-branching enzymes that simultaneously cleave short α-1,4 linked glucan chains and reattach them through an α-1,6 linkage to other chains within the starch molecule, thus creating a branched structure as well as increasing the number of non-reducing ends for further elongation by starch synthases. Disproportionating enzymes degrade short glucan chains to ultimately form glucose molecules that can be used for the synthesis of ADP-glucose or for the supply of energy for plant metabolism. Starch-debranching enzymes are believed to be involved in the trimming of the irregularly arranged glucan chains to maintain glucan branches in amylopectin molecules in a regular order.

Through whole-genome sequence analysis in rice, ten starch synthase genes have been identified (Hirose and Terao, 2004) and this is believed to represent the full set of starch synthase genes in higher plants. These genes are divided into five classes: the starch synthases I–IV (SSI, SSII, SSIII, and SSIV), and granule-bound starch synthase (GBSS) (Li *et al.*, 2003; Hirose and Terao, 2004). Their precise role individually and cooperatively in determining the final structure of the starch granule in wheat largely remains undefined although the roles of some starch synthase genes have been determined in different organs and different species.

Early published starch synthase genes in cereals encoding SSI, SSIIa, and SSIII in maize (Knight *et al.*, 1998; Harn *et al.*, 1998; Gao *et al.*, 1998), SSI, SSII, and SSIII in wheat (Li *et al.*, 1999*a*, 1999*b* and 2000), and SSI, and SSIIa in barley (Gubler *et al.*, 2000; Morell *et al.*, 2003) are homologous genes of SSI, SSIIa, and SSIIIa in rice (Hirose and Terao, 2004). The analysis of mutants of starch synthase genes in a wide range of species demonstrates that granule-bound starch synthase I (GBSSI) is critical for amylose biosynthesis (Ball *et al.*, 1996), but may also contribute to the synthesis of long chains of amylopectin (Maddelein *et al.*, 1994; Denyer *et al.*, 1996). In contrast, SSI, SSIIa, and SSIIIa are thought to be primarily involved in amylopectin synthesis and to contribute to the extension of specific subsets of available non-reducing ends within the molecule. Loss of SSIIa resulted in a decrease in chains with dp 11–30 in a number of different mutants, indicating that the SSIIa enzyme plays a role in extending shorter glucan chains (Craig *et al.*, 1998; Yamamori *et al.*, 2000; Umemoto *et al.*, 2002; Morell *et al.*, 2003; Zhang *et al.*, 2004; Konik-Rose *et al.*, 2007). Loss of SSIIIa in maize and barley confers a reduction in the proportion of very long chains (dp>50) and slightly reduced gelatinization temperature (Jane *et al.*, 1999; Li *et al.*, 2011; Lin *et al.*, 2011). *Arabidopsis* mutants defective for SSIVa seem to have fewer, larger starch granules within the plastid and a role in priming starch granule formation has been postulated for the SSIV protein (Roldan *et al.*, 2007). SSI mutants have been studied in *Arabidopsis* and rice. In transient storage starch of the *Arabidopsis* leaf, SSI is involved in biosynthesis of the small outer chains of the amylopectin cluster (dp 8–12) (Delvalle *et al.*, 2005). In the storage starch of rice endosperm, the SSI mutant had fewer short glucan chains (dp 8–12), and more intermediate glucan chains (dp 16–19) than the wild type (Fujita *et al.*, 2006, 2011).

To understand the role of SSI in the complex genetic background of hexaploid bread wheat, we have suppressed the expression of the *SSI* gene in wheat endosperm. A reduction of SSI protein in the soluble phase and inside starch granules in wheat endosperm was observed to produce several changes in the structure and properties of wheat starch including changes in the proportion of B-granules (<7 μM), the proportion of amylopectin glucan chains of dp 8–12, the content of amylose, an increased starch peak gelatinization temperature, and a reduced starch peak viscosity value.

## Materials and methods

### Generation of an RNAi construct to suppress wheat SSI

A plasmid cloning vector (pBx17IRcasNOT) was first constructed for the suppression of target sequences in wheat endosperm by RNAi technology. The vector was similar to that published by Zaplin *et al.* (2013) with some modifications. It contained two additional fragments. The first additional fragment included a forward oriented cassette of attR-*ccd*B (1447bp, Gateway cloning technology GibcoBRL/Life Technologies) with *Bam*HI site at 5′ site and *Eco*RI site at 3′ site, and a rice starch branching enzyme I (D10838) intron 4 (467bp from nucleotide 6202 to nucleotide 6668) in reverse orientation to the promoter, located between a HMW glutenin Bx17 promoter and a rice branching enzyme I intron 9 in forward orientation described by Zaplin *et al.* (2013). The second additional fragment was a reverse cassette of attR-*ccd*B (1435bp) with *Spe*I site at 5′ site and *Kpn*I at 3′ site between a rice branching enzyme I intron 9 and a NOS 3′ transcription terminator sequence described by Zaplin *et al.* (2013).

**Fig. 1.**
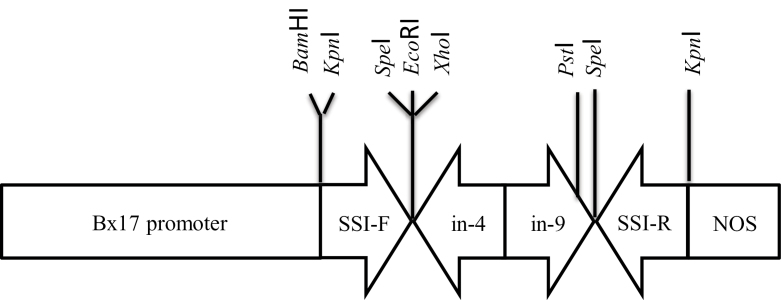
An SSI-RNAi construct for the suppression of SSI expression in wheat endosperm. The backbone was a pBC-SK plasmid (Stratagene, La Jolla, CA, USA). The abbreviations refer to: Bx17 promoter, a promoter sequence from the HMW glutenin Bx17 subunit sequence; SSI-F, a forward oriented SSI sequence from wheat SSI cDNA (574–1142bp, GenBank accession No. AF091803); in-4, an intron 4 sequence (507bp) of a rice starch branching enzyme I (GenBank accession No. D10838) in reverse orientation to the promoter; in-9, an intron 9 sequence (429bp) of a rice branching enzyme I in forward orientation; SS-R, a reverse complement of the wheat SSI cDNA sequence as above to the promoter; NOS, a NOS 3′ transcription terminator sequence (267bp) (Depicker *et al.*, 1982).

To create the SSI-RNAi construct, a target sequence (570bp) was amplified from wheat *SSI* cDNA (GenBank accession No. AF091803) by PCR using the primers SSI-IR-F 5′- AAAAGGATCCGGTA CCAGGGATTGCTGAGGATTCCATCG-3′ (containing *Bam*HI and *Kpn*I sites at 5′ end) and SSI-IR-R 5′- AAAAGAATTCA CTAGTCAGCAAGAAGGACTGGCACAAGG-3′ (containing *Eco*RI and *Spe*I sites at the 5′ end). PCR amplification was carried out under standard conditions using Hotstar polymerase (Qiagen, Australia) on a Hybaid PCR Express thermal cycler (Hybaid, UK). The thermal profile was 4min at 94 °C followed by 35 cycles of 30 s at 94 °C, 30 s at 58 °C and 1min at 72 °C. The resulting PCR fragments were first cloned in the reverse orientation into *Spe*I and *Kpn*I sites and then in the forward orientation into *Bam*HI and *Eco*RI sites to form a SSI-RNAi construct (Fig. 1).

### Transformation of wheat

Wheat (*Tritium aestivum* L. cv. Bob White 26) plants as a source of explant embryos were grown under containment glasshouse conditions (24 °C day and 18 °C night) under natural light. Embryos were transformed by biolistic bombardment as published (Pellegrineschi *et al.*, 2002). The SSI-RNAi construct was co-transformed with pCMneoSTLS2 (Maas *et al.*, 1997) encoding a selectable marker for neomycin phosphotransferase. After biolistic bombardment, calli were placed under selection on media containing 50mg l^–1^ geneticin (G418) and maintained at 24 °C in a 16h light (~25 μE m^–2^ s^–1^) and 8h dark regime. Calli were transferred onto fresh selection medium every three weeks. Plantlets were grown to 10cm high, transplanted into soil in 10cm pots, hardened on a misting bench for 2 weeks, and then transferred to a containment glasshouse with a temperature regime of 24 °C (day) and 18 °C (night). T_2_ plants were grown into soil in 10cm pots and T_3_ plants were grown with random design into soil in 20cm pots in the glasshouse described early.

Putative transgenic plants were screened for the presence of the transgene by PCR using the conditions described above. PCR primers Bx17-3′ 5′-CAACCATGTCCTGAACCTTCACC-3′, and SSI-IR-RM 5′-CTTGGCATTACAACCATCACACG-3′ were used for the amplification of a 360bp fragment specific to SSI-RNAi construct, including ~150bp Bx17 promoter and ~200bp of an *SSI* fragment in sense orientation.

###  RNA extraction and quantitative real-time PCR (qRT-PCR)

Total RNA from 15 days post-anthesis (DPA) developing endosperms was extracted following the procedure described in Clarke and Rahman (2005). The DNA-*free* ^TM^ kit (Ambion) was then used to remove contaminating DNA, following the manufacturer’s protocols. Where it was possible, the primers were designed to bind either side of an intron to enable detection of any genomic DNA contamination (Table 1). A total of 0.3 μg of RNA template was used for the cDNA synthesis in a 20 μl reaction at 75 °C for 10min for RNA denaturation, 50 °C for 50min for reverse transcription and 95 °C for 10min for enzyme inactivation. The reverse transcription reaction mix contained 4 μl of 5x Superscript reverse transcription PCR buffer (Invitrogen), 0.5 μl of dNTP mix (containing 10mM of each dNTP), 2.5 μl of 10mM DTT, 0.25 μl of RNasin (RNA guard) (40U μl^–1^) (Promega), 0.25 μl of 40 pmol μl^–1^ of polyT primer and 0.5 μl of SuperScript III reverse transcriptase (200U μl^–1^) (Invitrogen). For each PCR reaction (20 μl), 25ng of cDNA, 3.5mM MgCl_2_, 0.2mM of each dNTP, 5 pmol of primers, 1×SYBR Green I (Invitrogen), 1×PCR buffer (no MgCl_2_), and 0.25U of Platinum Taq DNA polymerase (Invitrogen) was used. The PCRs were conducted and analysed using a RotorGene 6000 (Corbett Life Science, Australia). The real-time PCR conditions were one cycle of 95 °C for 10min, 45 cycles of 95 °C for 20 s, 58 °C for 20 s, and 72 °C for 30 s, and melting from 72 °C to 95 °C rising by 1 °C for each step. Comparative quantitation analysis was used to calculate the comparative expression. The comparative expression for each genotype was calculated by mean values of the comparative concentration for mRNAs for *SSI* individually divided by the mean value of the comparative concentration for mRNAs for α-tubulin (GenBank accession No. DQ435663).

**Table 1.** qRT-PCR primers used for determination of RNA expression

Gene	Name	Oligonucleotide Sequence	Reference
*SSI*	SSIFw	AGGGTACAGGGTGGGCGTTCT	Sestili *et al.*, 2010
*SSI*	SSIR	GTAGGGTTGGTCCACGAAGG	Sestili *et al.*, 2010
Tubulin	ZLbTUB2F	AGTGTCCTGTCCACCCACTC	This study
Tubulin	ZLbTUB	CAAACCTCAGGGAAGCAGTCA	This study

### Analysis of starch synthase activities

Soluble proteins were extracted by homogenizing developing grains from 15 DPA on ice in an extraction buffer containing 50mM potassium phosphate buffer, pH 5, 5mM EDTA, 5mM DTT, 2 μl ml^–1^ protease inhibitor cocktail for plant cell and tissue extracts (Sigma, Australia) and 20% (v/v) glycerol followed by centrifugation at 13 000×g with microcentrifuge for 10min at 4 °C. The supernatant was decanted and the protein concentration was estimated using Coomassie Plus™ Protein Assay Reagent (Pierce, Australia). PAGE was carried out for the separation of proteins according to Laemmli (1970). Protein samples (20 μg) were loaded onto an 8% acrylamide gel containing 5mg ml^–1^ oyster glycogen (Sigma, Australia). Starch synthase activity in the gel was revealed by overnight incubation in an assay buffer containing 25mM Tris, 190mM glycine, 133mM (NH_4_)_2_SO_4_, 7.3mM MgCl_2_, 0.07% w/v BSA, 0.47% v/v β-mercaptoethanol and 1.24mM ADP-glucose, followed by staining of glucan chains with I_2_–KI solution containing 2g l^–1^ iodine and 20g l^–1^ potassium iodine (Li *et al.*, 2000).

### Analyses for grain properties and starch properties of T_3_ and T_4_ generations of mature grains

Analyses for grain weight (average seed weight was calculated from the total weight of 100 seeds), protein content, starch content, starch granules, starch swelling power, starch gelatinization temperature, and starch viscosity were performed on the T_3_ and T_4_ grains of two transgenic event-pairs (SSI-RNAi A and F) for the lines containing homozygous transgene from SSI-RNAi construct and their negative segregants which were derived from T_1_ heterozygous plants. T_3_ grains used for the analysis were from a single T_2_ plant of each of two transgenic event-pairs. To conduct the detail analysis, five T_3_ plants from each of two transgenic event-pairs were grown with random design to produce T_4_ grain for analysis.

### Grain weight

Grains from T_4_ SSI-RNAi and negative segregant lines were measured. Average grain weight produced was the mean values of triplicates of 100 grains for each of SSI-RNAi lines and their negative segregants.

### Assay of protein content

Protein content was measured by near infrared analysis instrument NIR 5000 (FOSS) as described previously (Cavanagh *et al.*, 2010).

### Assay of starch content

Wheat grains were first ground to wholemeal using a Cyclone mill machine using a sieve with a size of 0.5mm (Cyclote 1093, Tecator, Sweden). Starch content was assayed using AACC method 76.13 using 20mg of wholemeal for each of three replicate samples (Konik-Rose *et al.*, 2007).

### Isolation of starch

Starch was progressively eluted into water from a dough made from wholemeal flour of wheat grains. Starch was pelleted at 2000×g for 20min, resuspended in distilled water and pelleted again. Proteins were removed from the granule surface with 50 μL Proteinase K (1mg ml^–1^ in 25mM Tris–HCl, pH 7.5, 1mM EDTA) overnight at 37 °C. The starch was centrifuged for 20min at 2000×g then washed twice in distilled water, three times in 0.05% (w/v) sodium hydroxide followed by two washes in distilled water. Finally, the starch pellet was freeze dried under vacuum for two days.

### Assay of amylose content

The amylose content of starch was determined by a modification of the method of Morrison and Laignelet (1983). Lipid was first removed by dispersing 2mg (± 0.1mg) of starch in 1ml of 85% methanol and heating to 65 °C for 1h with occasional vortexing. After centrifugation at 13 000×g for 5min, the supernatant was removed and the extraction was repeated. The starch was dried at 65 °C for 1h and dissolved in 1ml urea dimethyl sulphoxide (UDMSO) solution (0.6M urea in 90% dimethyl sulphoxide). The mixture was immediately vortexed vigorously and kept in a 95 °C water bath for 1h with intermittent vortexing for complete dissolution of the starch. An aliquot of the starch–UDMSO solution (50 μl) was treated with 20 μl of I_2_–KI solution (1:10 w/w in water) and made up to 1ml with water. Sample absorbance of 200 μl solution was read with an E_max_ Precision Microplate Reader (Molecular Devices, USA) at 620nm along with standards containing amylose ranging from 0–100%, made from combined potato amylose and corn amylopectin (Sigma). Absorbance values were converted to percentage amylose using a regression equation derived from the standard samples.

### Analysis of starch granule size

Granule size distribution (by volume) of the starch slurries was determined using a laser diffraction particle size analyser (Mastersizer 2000, Malvern Instruments, Malvern, England). The percentage of small B-type starch granules was determined using a cut-off diameter of 7 μm (refer to the result section). The diameter of A-type starch granules was calculated as the diameter of starch granules at the peak of A-type starch granules.

### Analysis of starch-granule-bound proteins of mature grains

Starch-granule-bound proteins were isolated and separated on SDS–PAGE gel as described previously (Rahman *et al.*, 1995). The proteins were then stained by silver staining (Li *et al.*, 1999a) or Sypro Ruby Protein Gel Stain (Invitrogen). The protein gels were scanned to image files (Epson Perfection 2450 PHOTO; Epson America Inc., CA, USA).

### Analysis of starch chain length distribution

The starch for each sample was weighed into 20mg triplicates and debranched by iso-amylase (Megazyme, Ireland). The chain length distribution of amylopectin was analysed according to O’Shea *et al.* (1998), on a P/ACE 5510 capillary electrophoresis system (Beckman Coulter, Australia).

### Analysis of starch gelatinization properties

Starch gelatinization parameters were measured in a Pyris 1 differential scanning calorimeter (Perkin Elmer, Norwalk, CT). Starch was combined with water in a 1:2 (w/v) ratio. This mixture (40–50mg, accurately determined) was sealed in a stainless steel pan and scanned at 10 °C min^–1^ from 20 °C–140 °C with an empty stainless steel pan as a reference. The PYRIS software was used to calculate gelatinization temperatures and enthalpy.

### Measurement of swelling power

Swelling power was measured by the 40mg swelling test (Konik-Rose *et al.*, 2001). Briefly, approximately 40mg of purified starches (accurately determined) were gelatinized in 1ml of water at 92.5 °C for 30min with regular inversions. Solutions were cooled for 10min in a waterbath at room temperature. Samples were centrifuged at 13 000×g for 10min. The supernatants were removed and the residues were weighed. Swelling power was calculated as the weight of residue divided by the dry weight of starch.

### Rapid viscosity analysis

Pasting properties were measured using a Rapid Visco Analyzer (RVA Model 3D+, Newport Scientific Pty Ltd., NSW, Australia). Starch (3.0g) was added to distilled water (25.0ml) in the Rapid Visco Analyzer pan and analysed under the conditions of continuous shear (160rpm). The rapid viscosity analysis (RVA) run profile was: heat at 50 °C for 2min, heat to 95 °C over 6min, hold at 95 °C for 4min, cool to 50 °C over 4min, and hold at 50 °C for 4min. The measured parameters were: peak viscosity (PV) at 95 °C, holding strength (HS) at the end of a 95 °C holding period, breakdown (BD) = PV–HS, final viscosity (FV) at the end of a 50 °C holding period, setback (SB) = FV–HS, pasting temperature (PT) at onset of viscosity, and peak temperature (Pt) at PV. The software Thermocline for Windows version 2.2 (Newport Scientific Pty Ltd., NSW, Australia) was used for collection and analysis of data.

### Morphology and crystalline structure of starch granules

Starch granule morphology was examined with a scanning electron microscope (ZEISS EVO LS15). Purified starches were sputter-coated with gold and scanned at 20kV at room temperature at a ×300 or ×1000 magnification.

For examination of the crystalline structure, starch granules were mounted in water and observed by light microscopy (Leica-DMR) with crossed polarized filters to reveal birefringence in the starch granules at a ×400 magnification.

### Statistical analyses of grain components or starch properties

Statistical analyses were performed using Genstat version 13. Analysis of variance was performed for grain weight, starch content, amylose content, protein content, B starch granule, swelling power, RVA data, differential scanning calorimetry (DSC) data, and quantitative RT-PCT data to obtain the least significant difference (LSD, *P<*0.05), looking at changes between two transgenic event-pairs.

## Results

### Production of SSI-RNAi transgenic wheat plants

**Fig. 2.**
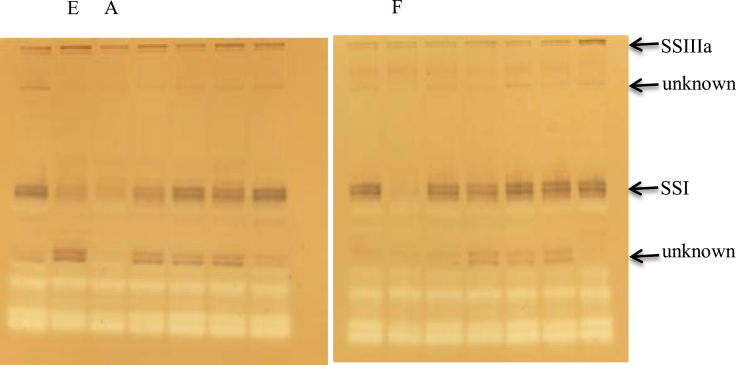
Zymogram analysis of wheat endosperm extracts from T_1_ SSI-RNAi transgenic lines at 15 DPA, separated by non-denaturing PAGE. Three lines with reduced SSI enzyme activity are labelled above the lines. Other lines, which are without obvious changes or have lower reduction of SSI enzyme activities, are not labelled. The activities of SSI, SSIII, and unknown proteins are indicated on the right side. A, SSI-RNAi A; E, SSI-RNAi E; F, SSI-RNAi F.

Three independent SSI-RNAi suppressed lines were produced by particle bombardment. To obtain transgenic wheat plants, 800 wheat embryos were bombarded with the SSI-RNAi construct (Fig. 1) and selected on media containing G418. To ensure independent transgenic events, only a single shoot was regenerated from a single callus. In total, thirty transgenic events were identified by PCR amplification of an SSI-RNAi construct-specific DNA fragment. To identify SSI-RNAi suppressed lines, the expression of SSI enzymatic activity in developing T_1_ endosperm of SSI-RNAi construct transgenic lines was analysed by native polyacrylamide activity gels (zymogram). SSI and SSIII in these zymograms were identified immunologically as in previous studies (Cao *et al.*, 1999; Li *et al.*, 2000). Three independent SSI-RNAi suppressed lines (SSI-RNAi A, E, and F) were identified with a noticeably reduced SSI enzyme activity (Fig. 2). These lines were selfed to create T_2_ plants, and homozygous transgenic and negative segregant T_3_ SSI-RNAi A and SSI-RNAi F lines were analysed by PCR to identify whether the SSI-RNAi transgene was present or absent in all twenty of the T_3_ transgenic plants originating from one T_2_ transgenic plant. SSI-RNAi line E was not stable and was not studied further.

### Amounts of granule-bound proteins in SSI-RNAi transgenic wheat plants

**Fig. 3.**
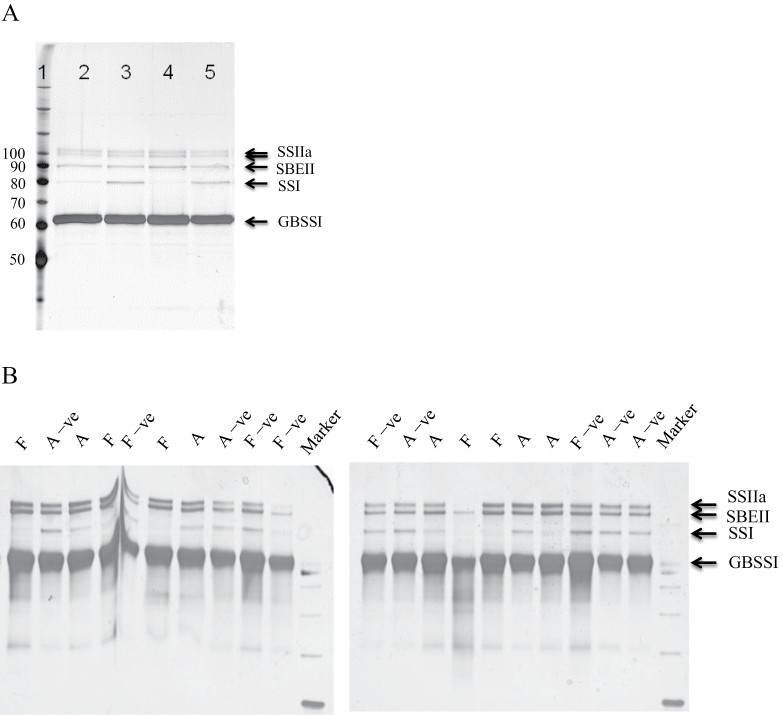
Starch granule proteins of mature wheat grains from T_3_ (in A) and T_4_ generation (in B) SSI-RNAi suppressed lines and their negative segregants are revealed by silver staining (in A) or Sypro Ruby Protein Gel Stain (Invitrogen) (in B). (A) Grain starch from one T_3_ plant from each of two transgenic event-pairs was analysed. Lane 1, benchmark protein ladder (Invitrogen, Carlsbaad, CA, USA); Lane 2, SSI-RNAi A; Lane 3, SSI-RNAi A negative segregant; Lane 4, SSI-RNAi F; Lane 5, SSI-RNAi F negative segregant. (B) Grain starch was analysed from five T_4_ plants for each of two transgenic event-pairs. SSI-RNAi A (named as A in lanes 3, 7, 14, 17, 18); SSI-RNAi A negative segregant (named as A –ve in lanes 2, 8, 13, 20, 21); SSI-RNAi F (named as F in lanes 1, 4, 6, 15, 16); and SSI-RNAi F negative segregant (named as F –ve in lanes 5, 9, 10, 12, 19). Size of protein markers is indicated on the left and names of proteins are labelled on the right. Marker is the protein marker.

A reduced amount of SSI protein inside grain starch granules was observed in wheat SSI-RNAi suppressed lines. A portion of the starch-biosynthetic enzymes is present inside starch granules of mature wheat grain, where they are trapped during starch synthesis. The major granule-bound proteins are GBSSI, SSIIa, SBEIIa, and SSI (Fig. 3A). To confirm that homozygous SSI-RNAi transgenic lines contained reduced levels of SSI protein compared with their negative segregants, proteins were extracted from the same amount of starch (4mg) and separated by SDS–PAGE and detected by silver staining (T_3_ grain) (Fig. 3A) or SyproRuby staining (T_4_ grain) (Fig. 3B). In both T_3_ SSI-RNAi lines A and F there was a clear reduction in SSI protein, whereas the other granule-bound proteins (GBSSI, SSIIa, and SBEIIa) were unchanged (Fig. 3A lanes 2 and 4, respectively). In individual T4 grains, the abundance of granule-bound SSI protein was also reduced but this was more apparent for SSI RNAi line F than line A (Fig. 3B).

### Expression of mRNAs for SSI gene

**Fig. 4.**
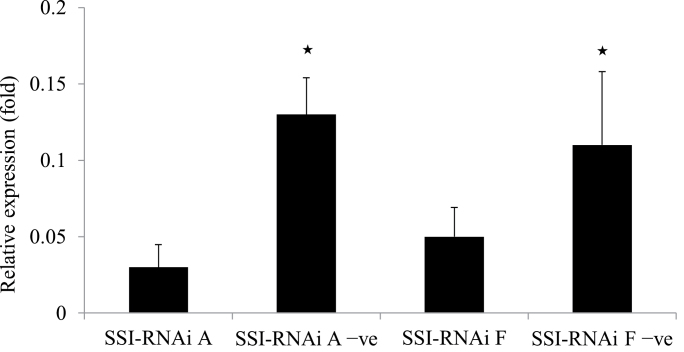
Relative expression of mRNAs of *SSI* in developing endosperms at 15 DPA from the T_4_ generation of SSI-RNAi suppressed lines and their negative segregants. The names of lines are labelled underneath the columns (–ve; negative segregant). Total RNAs from five plants were used for each of two transgenic lines and their negative segregant lines. *Tubulin* gene was used as a reference gene. * indicates the significant difference at *P*<0.05.

The SSI-RNAi transgene changes the levels of expressed mRNAs for *SSI* measured by quantitative real-time PCR. Relative expression data showed that the level of *SSI* mRNA in T_4_ immature endosperms of both SSI-RNAi lines was significantly lower than that from their negative segregants (Fig. 4).

### Properties of the mature transgenic grain

To determine the effect of the suppression of SSI expression in wheat grain, analysis of grain weight and its composition was undertaken in T_3_ (Table 2A) and T_4_ (Table 2B) generation grains. Grain weight was similar for all lines for both SSI-RNAi event-pairs (i.e. homozygous vs. negative segregant) at approximately 51–53mg per grain for T_3_ grains and 57–61mg per grain for T_4_ grains. Overall, both SSI-RNAi event-pairs were also similar in their protein contents (approximately 16–20% for T_3_ grains and 15–16% for T_4_ grains) and starch contents (approximately 56–64% for T_3_ grains and 57–62% for T_4_ grains).

### Properties of endosperm starch

#### Amylose content and percentage of B starch granules

Apparent amylose content of the starch is affected by the suppressed SSI expression in wheat endosperm. The results, as measured by iodine staining, showed 4% and 8% more amylose in SSI-RNAi A and SSI-RNAi F than that from their negative segregants in T_3_ grains, respectively (Table 2A). In T_4_ grains, amylose content did not change in the SSI-RNAi A line and significantly increased in the SSI-RNAi F line by 3.5% to 30.9% (*P*<0.05) (Table 2B). Thus, the SSI-RNAi F line contained significantly less amylopectin compared with the other three lines.

**Table 2.** Grain weight and composition of SSI-RNAi and negative segregant lines A. T_3_ SSI-RNAi and negative segregant lines

Line	Grain weight (mg)	Protein content (%)	Starch content (%)	Amylose content (%)
SSI-RNAi A	51.6 (6.8)	19.3 (1.5)	56.5 (2.9)	33.9 (2.6)
SSI-RNAi A –ve	53.7 (6.7)	20.7 (1.8)	56.6 (1.5)	29.5 (1.4)
SSI-RNAi F	51.6 (3.7)	16.8 (1.5)	63.5 (0.6)	33.7 (1.7)
SSI-RNAi F –ve	51.0 (4.8)	16.4 (1.3)	62.8 (3.9)	25.8 (5.2)

Protein content, starch content and β-glucan content are expressed as a percentage of grain weight and are the mean of three technical replicates. Amylose content is expressed as a percentage of total starch. –ve: denotes the negative segregant of SSI-RNAi A or F transgenic line. Standard deviation in brackets.

**Table:** B. T_4_ SSI-RNAi and negative segregant lines

Lines	Grain weight (mg)	Protein content (%)	Starch content (%)	Amylose content (%)	Amylopectin content (%)	B starch granule (%)
SSI-RNAi A	61.1 (3.5)^a^	16.3 (1.0)^a^	57.6 (1.3)^a^	28.9 (2.6)^b^	71.1 (2.6)^a^	15.2 (2.3)^b^
SSI-RNAi A –ve	60.5 (2.6)^a^	15.4 (1.5)^a^	62.3 (3.8)^a^	28.5 (1.9)^b^	71.5 (1.9)^a^	16.1 (1.1)^a^
SSI-RNAi F	57.5 (4.3)^a^	15.3 (1.1)^a^	57.1 (3.7)^b^	30.9 (2.0)^a^	69.1 (2.0)^b^	14.7 (1.3)^b^
SSI-RNAi F –ve	60.4 (2.8)^a^	15.0 (2.4)^a^	60.2 (5.1)^a^	27.4 (1.7)^b^	72.6 (1.7)^a^	17.7 (1.3)^a^
LSD (*P*<0.05)	4.5	2.2	5.0	2.8	2.8	2.1

Protein content and starch content are expressed as a percentage of grain weight and are the mean of three technical replicates. Amylose content and B starch granule are expressed as a percentage of total starch. –ve: denotes the negative segregant of SSI-RNAi A or F transgenic line. Numbers are mean values from five plants for each of two transgenic lines and their negative segregants. Standard deviation presents in brackets. The letters a or b next to values are based on LSD; mean values with the same letter are not statistically significantly different (*P*<0.05), whereas those with a different letter are statistically significantly different.

**Fig. 5.**
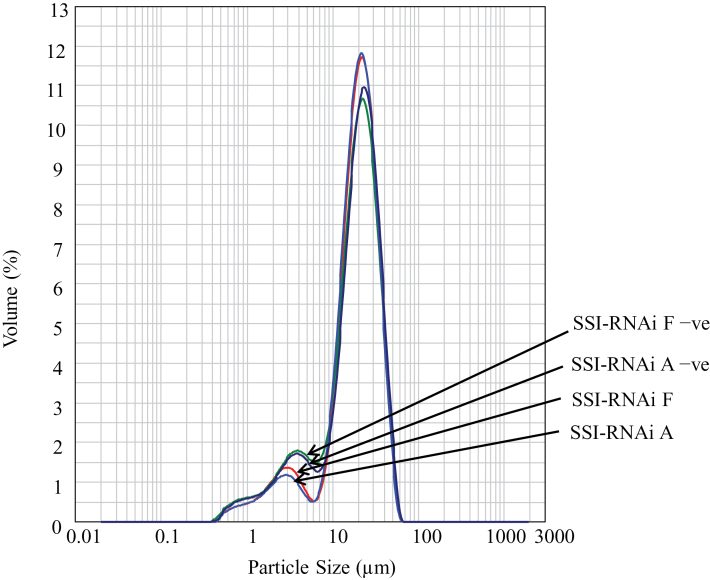
Particle size analysis of grain starch granules of T_4_ SSI-RNAi and negative segregant lines. The *y* axis expresses the amount of each size of starch granules as a percentage of total starch. The *x* axis represents the size of starch granules. The names of lines are labelled on the right of the micrograph.

The suppression of SSI expression also changes the sizes of starch granules in wheat grains. Starch granule size distribution was analysed with a laser particle analyser, which clearly showed two peaks with a minimum around 7 μm (Fig. 5). By convention the more abundant larger granules are designated A granules and the smaller B granules were here defined as those smaller than 7 μm in diameter. The results showed that in T_4_ grains, SSI-RNAi A and SSI-RNAi F contained a significantly lower percentage of B-granules (15.2% and 14.7%, respectively) than that from their negative segregant lines (16.1% and 17.7%, respectively; *P*<0.05) (Table 2B).

#### Chain length distribution

**Fig. 6.**
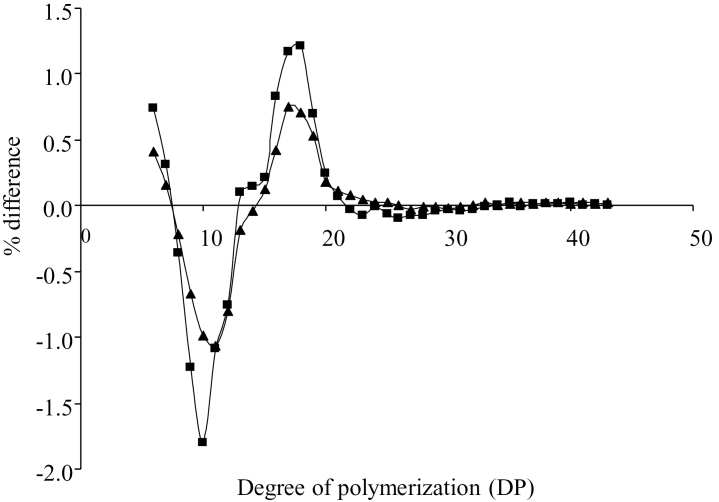
Chain length distribution of amylopectin of wheat grain starches from T_4_ generation of SSI-RNAi suppressed lines and their negative segregants. Difference plot of chain lengths was calculated as: mean values of chain lengths for negative segregant lines subtracted from mean values of chain lengths for the SSI-RNAi lines. Negative values indicate that the SSI-RNAi lines contained fewer chains of a given length and *vice versa*. The two difference plots are SSI-RNAi A lines – SSI-RNAi A negative segregant lines (triangles), and SSI-RNAi F lines – SSI-RNAi F negative segregant lines (squares). Starches from five plants were used from each of two transgenic lines and their negative segregant lines.

The suppression of SSI expression in wheat grain modifies the chain length distribution of grain starch. To reveal the glucan chain length distribution of amylopectin in the SSI-RNAi suppressed T_4_ SSI-RNAi A and SSI-RNAi F lines, purified starches were debranched and the glucan chains were separated by capillary electrophoresis. The relative proportion of the total for each glucan chain length was determined. The mean value (starches from five plants for each of two transgenic lines and their negative segregant lines) for each amylopectin glucan chain of the negative segregant line was subtracted from the corresponding value for the SSI-RNAi line. The resulting differences in chain length distribution between the SSI-RNAi transgenic lines and their negative segregants are shown in Fig. 6. Amylopectin molecules from the transgenic SSI-RNAi lines had fewer short chains (dp 8–12) and more chains of dp 6–7 and medium length chains (dp 13–22) than the negative segregants. Glucan chains greater than dp 25 seem to be unaffected by SSI suppression. This phenotype was more severe in line SSI-RNAi F, and this correlated with the reduction in SSI protein (it is a negative correlation with protein level).

#### Starch gelatinization temperature

The reduction of SSI proteins in the SSI-RNAi transgenic lines leads to the changes of starch gelatinization. Starch from T_4_ grains of both SSI-RNAi lines had significantly higher peak gelatinization temperature and end gelatinization temperature (*P*<0.05, Table 3) than that from their negative segregants. However, all had similar onset gelatinization temperatures. SSI-RNAi F starch had both peak and end gelatinization temperatures higher than that from the SSI-RNAi A line, but the difference was not significant for peak gelatinization temperature. The SSI-RNAi F line had significantly lower enthalpy (DeltaH) for gelatinization temperature peak than that from the negative segregant and SSI-RNAi A line (*P*<0.05). With the amylose-lipid dissociation peak, there were no significant differences for the dissociation temperature and the DeltaH values between the SSI-RNAi A line or SSI-RNAi F line and their negative segregants (*P*<0.05).

**Table 3.** Differential scanning calorimetry measurements of starches from T_4_ generation of SSI-RNAi lines and control lines

Line	Starch gelatinization peak				Amylose-lipid dissociation peak			
Onset	Peak	End	DeltaH	Onset	Peak	End	DeltaH
SSI-RNAi A	58.0^a^	61.5^a^	67.3^b^	4.5^a^	98.2^a^	104.6^a^	110.0^a^	0.6^a^
SSI-RNAi A –ve	57.6^a^	60.5^b^	65.5^c^	4.7^a^	98.9^a^	104.7^a^	110.4^a^	0.5^a^
SSI-RNAi F	58.0^a^	62.2^a^	68.5^a^	4.1^b^	98.2^a^	104.4^a^	108.8^a^	0.7^a^
SSI-RNAi F –ve	57.6^a^	60.8^b^	65.7^c^	4.6^a^	97.2^a^	104.2^a^	109.8^a^	0.6^a^
LSD (*P*<0.05)	0.9	0.8	0.7	0.4	2.0	0.6	1.7	0.2

DSC was performed in duplicates on starches from five lines of two event-pairs. –ve denotes the negative segregant of SSI-RNAi A or F transgenic line. Numbers are mean values from five plants for each of two transgenic lines and their negative segregants. The letters a, b, or c next to values are based on LSD; mean values with the same letter are not statistically significantly different (*P*<0.05), whereas those with different letters are statistically different.

#### Swelling power

The reduction of SSI protein in SSI-RNAi transgenic lines reduces starch-swelling power. A 40mg swelling-power test was conducted on grain starches isolated from five plants of each of two SSI-RNAi transgenic lines and their negative segregant lines. The negative segregant control starches absorb approximately 12 times their weight of water, whereas starch from the T_4_ grains of SSI-RNAi A and SSI-RNAi F absorb only 11 or 10 times their weight, a statistically significant reduction of 8% or 20% respectively (*P*<0.05; Table 4).

**Table 4.** Swelling power and rapid viscosity analysis measurements of starches from the T_4_ generation of SSI-RNAi lines and their control lines

Line	Swelling power	RVA						
		PV (RVU)	HS (RVU)	BD (RVU)	FV (RVU)	SB (RVU)	Pt (min)	PT (°C)
SSI-RNAi A	11.0^b^	188.3^b^	150.1^b^	38.3^a^	265.7^b^	115.6^b^	10.1^a^	87.7^a^
SSI-RNAi A –ve	12.0^a^	222.7^a^	187.7^a^	37.1^a^	338.5^a^	150.9^a^	10.0^a^	85.8^b^
SSI-RNAi F	10.0^b^	167.8^b^	130.6^c^	37.3^a^	231.2^b^	100.6^b^	10.1^a^	88.9^a^
SSI-RNAi F –ve	12.7^a^	221.7^a^	179.8^a^	41.9^a^	329.0^a^	149.3^a^	9.9^a^	86.1^b^
LSD (*P*<0.05)	0.6	23.6	19.0	6.3	36.1	17.5	0.2	1.6

Swelling power and RVA was performed in duplicates on starches from five lines of two event-pairs. –ve denotes the negative segregant of SSI-RNAi A or F transgenic line. Numbers are mean values from five plants for each of two transgenic lines and their negative segregants. The letters a or b next to values are based on LSD; mean values with the same letter are not statistically significantly different (*P*<0.05), whereas those with a different letter are statistically significantly different. PV: peak viscosity, HS: holding strength, BD: breakdown (BD=PV–HS), FV: final viscosity, SB: setback (SB=FV–HS), Pt: peak temperature, PT: Pasting temperature.

#### Rapid viscosity analysis

The suppression of SSI protein in SSI-RNAi transgenic lines changes the viscosity properties of starch. Rapid viscosity analysis (RVA) showed that starch from T_4_ grains of SSI-RNAi A and SSI-RNAi F lines had significantly lower peak viscosity (PV), holding strength (HS), final viscosity (FV), setback (SB), and higher pasting temperature (PT) than that from their negative segregants (*P*<0.05). The value for HS was lower from the SSI-RNAi F line than that from the SSI-RNAi A line (Table 4).

#### Starch granule morphology

**Fig. 7.**
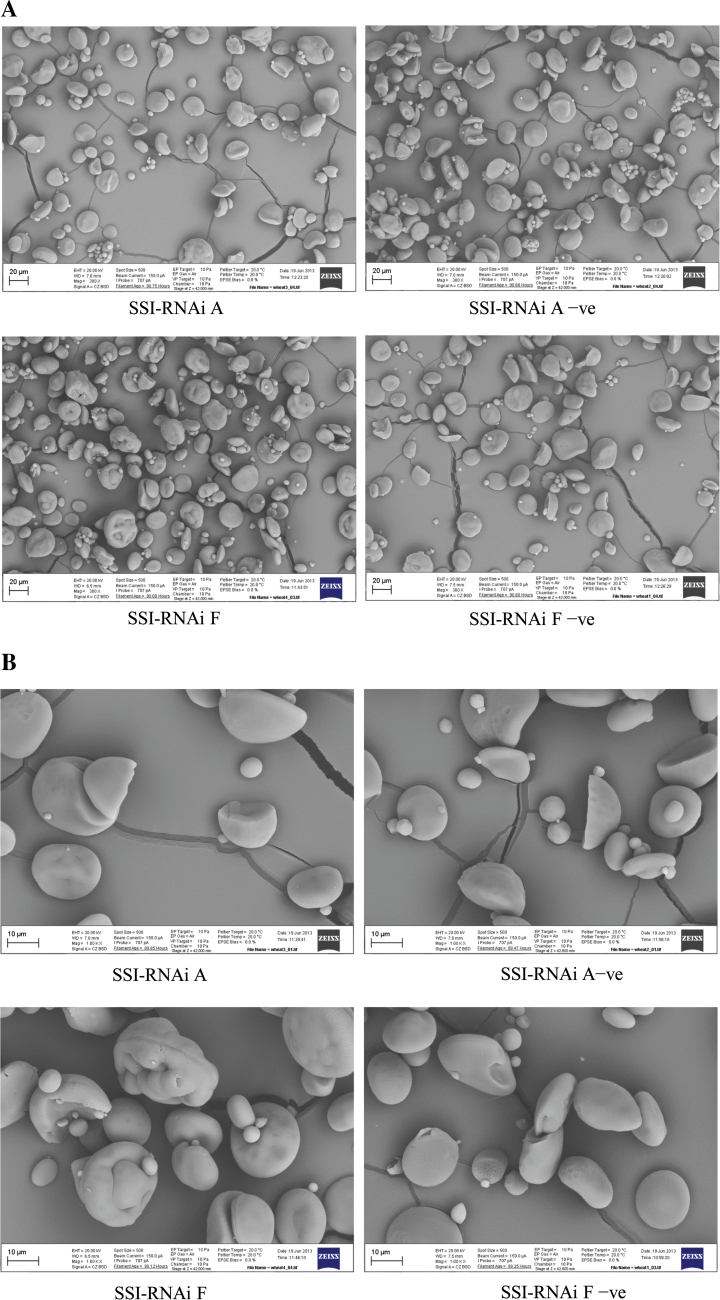
Scanning electron micrographs of wheat grain starches from T_4_ generation of SSI-RNAi suppressed lines and their negative segregants. The names of lines are labelled underneath the micrographs. (A) ×300 magnification. (B) ×1000 magnification. Bars indicating 20 μm (A) or 10 μm (B) are shown on each micrograph.

Changes in starch granule morphology of the SSI-RNAi lines were examined by scanning electron microscopy. There were no obvious changes between starch granules from the SSI-RNAi A line and its negative segregant under ×300 (Fig. 7A) and ×1000 (Fig. 7B) magnifications. However, a large number of abnormal starch granules were observed in the SSI-RNAi F line under ×300 magnification (Fig. 7A), and at higher magnification (×1000) the larger granules especially appeared to be of a compound nature and to have a distorted surface compared with the smooth granules of the negative segregant.

#### Birefringence

**Fig. 8.**
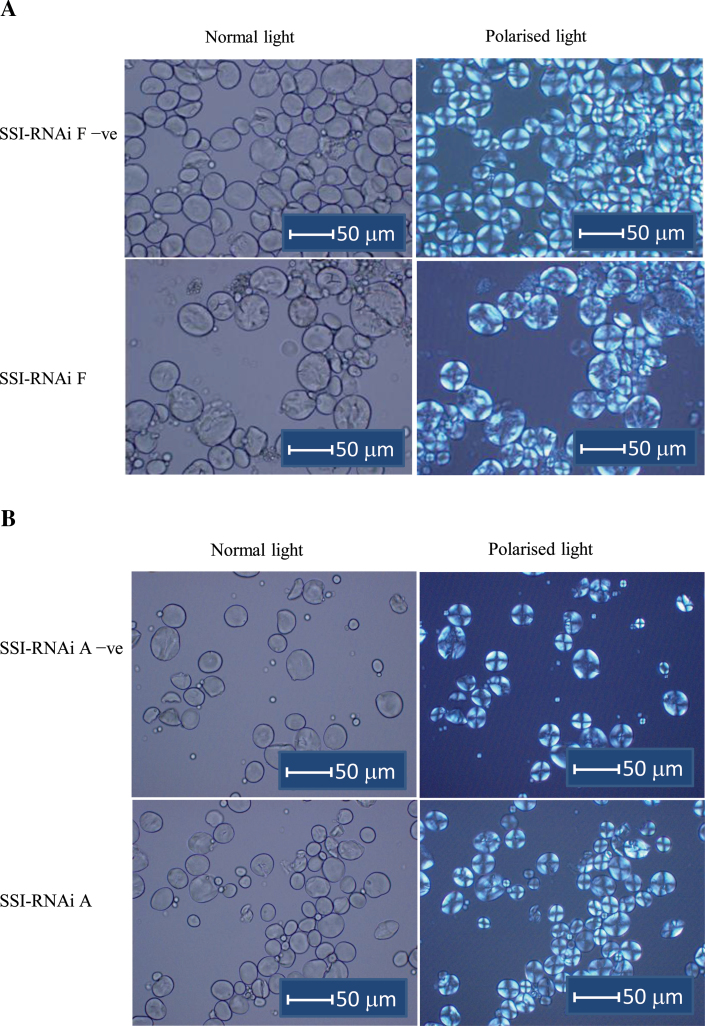
Micrographs of wheat grain starches from T_4_ generation of SSI-RNAi suppressed lines and their negative segregants (at ×400 magnification). The names of lines are labelled on the left side of the micrographs. Types of light are indicated on the top of the micrographs. (A) SSI-RNAi F and its negative segregate. (B) SSI-RNAi A and its negative segregate. Bars indicating 50 μm are shown on each micrograph.

The suppression of SSI protein in the SSI-RNAi transgenic lines changes the crystalline order in the starch granules. Birefringence provides a visual measure of the level of crystalline order in starch granules. Spherical-shaped granules show a distinct Maltese cross when examined by polarized light (Fig. 8A). Starch granules from the SSI-RNAi A line had a normal Maltese cross, and a similar brightness compared with the negative segregant (Fig. 8A). However, starch granules from the SSI-RNAi F line showed some disruption in clarity of the Maltese cross and a loss of brightness compared with the negative segregant. In a few normal-shaped granules, a clear Maltese cross was visible; however in the majority of the granules, only the points of the cross near the edges of the granules could be seen and the centre of the granules appeared dull and misshapen (Fig. 8B). B-starch granules that were present were not deformed in shape and did show a Maltese cross indicating that they possessed a level of structural order.

#### Amounts of protein, starch, amylose, and amylopectin on a per caryopsis basis

Expressing grain composition data on a percentage basis does not reflect the underlying synthesis rate of starch in wheat grain. To examine the absolute levels of protein and starch synthesis in the SSI-RNAi lines and their negative segregants, contents were calculated on a per caryopsis basis (Table 5). Such analysis revealed that the observed increase of amylose content in the SSI-RNAi F line was due to a reduction of amylopectin synthesis, and not the increase of amylose synthesis. The reduction of amylopectin synthesis was also reflected in the reduction of starch content. There were no changes for the synthesis of protein content on a per caryopsis basis.

**Table 5.** Amount of protein and starch of T_4_ SSI-RNAi and negative segregant lines on a per seed basis

Lines	Protein content (mg)	Starch content (mg)	Amylose content (mg)	Amylopectin content (mg)
SSI-RNAi A	9.9 (0.5)^a^	35.2 (2.7)^a^	10.1 (0.7)^a^	25.1 (2.6)^a^
SSI-RNAi A –ve	9.4 (1.1)^a^	37.8 (3.7)^a^	10.7 (0.9)^a^	27.0 (3.2)^a^
SSI-RNAi F	8.8 (1.2)^a^	32.8 (3.2)^b^	10.2 (1.6)^a^	22.6 (1.7)^b^
SSI-RNAi F –ve	9.1 (1.6)^a^	36.3 (2.5)^a^	9.9 (1.1)^a^	26.4 (1.7)^a^
LSD (*P*<0.05)	1.6	4.1	1.5	3.2

–ve denotes the negative segregant of SSI-RNAi A or F transgenic line. Numbers are mean values from five plants for each of two transgenic lines and their negative segregants. Standard deviation present in brackets. The letters a or b next to values are based on LSD; mean values with the same letter are not statistically significantly different (*P*<0.05), whereas those with a different letter are statistically significantly different.

## Discussion

### Reduction of the SSI protein by RNAi suppression

Two independent SSI-RNAi transgenic events (SSI-RNAi A and F) showed that insertion of the SSI-RNAi construct into the wheat genome conferred a clearly altered phenotype upon wheat endosperm starch. The RNAi technology has been widely used for the suppression of endogenous genes in cereal and oil grains (Liu *et al.*, 2002; Regina *et al.*, 2006) since it was first discovered (Smith *et al.*, 2000). Changes in the endosperm starch of the SSI-RNAi transgenic lines in this work included an increase in the abundance of very short chains (dp 6 and 7) and intermediate chains (dp 13–22), and a reduction in the abundance of short chains (dp 8–12) of amylopectin. The altered amylopectin chain-length distribution co-segregated with the SSI-RNAi transgene, and with the reduction of the SSI protein in the soluble phase and bound to the starch granule in both lines. Therefore, we interpret this to mean that the SSI-RNAi transgene is responsible for the suppression of the native level of SSI protein that is causing the changes in the distributions of the starch chain length.

The SSI enzymatic activity in the endosperm was clearly reduced as measured by zymogram analysis, and it seems that this limited the extension of the very short chains of dp 6–7 so that these chains increased in abundance while the glucose chains of dp 8–12 were reduced. Thus, we conclude that the very short glucose chains of dp 6–7 residues are the substrates for SSI enzyme, which extends them to a length of 8–12 glucose residues within the amylopectin clusters. The effects of SSI on the chain length distribution are consistent with the role of SSI activities in rice grains (Fujita *et al.*, 2006, 2011) and *Arabidopsis* leaves (Delvalle *et al.*, 2005). It was hypothesized that loss of SSI leaves more reducing ends available to SSII, and SSII is able to generate the longer dp 12–18 chains, whereas SSI does not elongate chains beyond dp 12 (Delvalle *et al.*, 2005). Thus, SSI has a similar role in the synthesis of starch in dicot plants (e.g. *Arabidopsis*) as well as monocot plants (e.g. rice and wheat).

### Reduction in SSI protein causes changes to starch morphology and composition, and starch functional properties in wheat endosperm

In the line with the largest decrease in SSI protein (SSI-RNAi F), a large number of abnormal starch granules were detected under high magnification with a scanning electron microscope. The SSI-RNAi F line also had an altered birefringence pattern with an abnormal less bright Maltese cross. Changes in starch granule morphology and crystallinity were not detected in SSI mutants of rice endosperm starch and *Arabidopsis* leaf starch, although in the latter, starch granules were slightly elongated (Delvalle *et al.*, 2005; Fujita *et al*., 2006). In comparison with the suppression of SSI, the suppression of SSIIa or SBEIIa expression in mutants of pea, maize, wheat, and barley cause more severe changes in starch granule structure with many deformed granules, and nearly complete loss of birefringence for all starch granules (Wang *et al.*, 1993; Craig *et al.*, 1998; Yamamori *et al.*, 2000; Morell *et al.*, 2003; Regina *et al.*, 2006; Konik-Rose *et al.*, 2007).

An increase in apparent amylose content and a reduced proportion of B starch granules were observed in the SSI-RNAi F line. The increase of amylose content was not due to an increase of amylose synthesis, but due to the decrease of amylopectin synthesis on a per caryopsis basis. Such changes in amylose content were not observed in rice endosperm (Fujita *et al*., 2006). However, in *Arabidopsis* leaf, amylose content was also increased and there were more small starch granules (Delvalle *et al.*, 2005). In comparison with the SSI-RNAi F line, not only the wheat and barley SSIIa mutants, but also the wheat SBEIIa suppression line or the barley SBEIIa and SBEIIb suppression line contain much higher amylose content (~45% in wheat SSIIa mutant, and ~70% or more for others) (Yamamori *et al.*, 2000; Morell *et al.*, 2003; Konik-Rose *et al.*, 2007; Regina *et al.*, 2006, 2010, 2012).

The reduction of starch swelling power in the wheat SSI-RNAi lines may be due to the reduction of the frequency of short chains of amylopectin (dp 8–12), as the SSI-RNAi A line, which has similar amylose content as its negative segregant line, had lower swelling power than that of its negative segregant line. The reduction of starch swelling power was also reported for starch containing increased amylose content through the suppression of SBEIIa in barley (Regina *et al.*, 2010), SSIIa in wheat (Konik-Rose *et al.*, 2007), or through the increase of functional alleles of GBSSI (Yamamori & Quynh, 2000). The suppression of SSI in wheat had less effect on starch swelling power than that observed from the suppression of SSIIa expression in barley and wheat (Morell *et al.*, 2003; Konik-Rose *et al*., 2007). The level of the reduction of starch swelling power in wheat SSI-RNAi lines is similar to that from single null mutation of SSIIa in wheat, and much lower than that from double null and triple null SSIIa mutations (Konik-Rose *et al.*, 2007), probably owing to the lower increase in amylose content in the SSI-RNAi lines.

Both SSI-RNAi lines showed decreased starch peak viscosity, decreased starch final viscosity, increased starch peak gelatinization temperature, and increased starch end gelatinization temperature compared with their negative segregants. This may be due to their starch containing less short chains (dp 8–12) and more intermediate chains (dp 13–22). Low peak viscosity and high peak gelatinization temperature were also observed in rice SSI mutant endosperm starch (Fujita *et al*., 2006; Delvalle *et al.*, 2005). As SSI protein is found in the soluble phase and bound to the starch granule, and both forms are reduced in abundance in the RNAi lines, both could be important in controlling starch morphology, composition, and functional properties in wheat endosperm. However, further work is required to make definitive conclusions about the relative contribution of each fraction.

In conclusion, this work showed that starch synthase I in wheat grains can be effectively suppressed using RNAi technology, that when SSI is sufficiently suppressed the abundance of SSI in the soluble phase and bound to the starch granule is noticeably reduced and that this changes the starch structure, granule morphology, and starch properties. SSI clearly plays an important role in biosynthesis of wheat endosperm storage starch, but more work is required to determine the relative importance of SSI in the granule stroma and granule-bound forms.

## Abbreviations:

DSC: differential scanning calorimetry
SSI: starch synthase I
PCR: polymerase chain reaction
RS: resistant starch
GI: glycaemic index.
